# Identification of Appropriate Reference Genes for Human Mesenchymal Cells during Expansion and Differentiation

**DOI:** 10.1371/journal.pone.0073792

**Published:** 2013-09-02

**Authors:** Paola Romina Amable, Marcus Vinicius Telles Teixeira, Rosana Bizon Vieira Carias, José Mauro Granjeiro, Radovan Borojevic

**Affiliations:** 1 Excellion Biomedical Services, Petrópolis, Rio de Janeiro, Brazil; 2 Bioengineering, National Institute of Metrology, Quality and Technology, Rio de Janeiro, Brazil; Rutgers - New Jersey Medical School, United States of America

## Abstract

**Background:**

Quantitative real time polymerase chain reaction (qPCR) is an extremely powerful technique for monitoring gene expression. The quantity of the messenger ribonucleic acids (mRNA) of interest should be normalized using a reference gene, in order to avoid unreliable results originated by the obtained RNA quality and quantity, manipulation errors and inhibitory contaminants. A reference gene is any gene that is stably and consistently expressed under the conditions being studied. Completely false data can be generated if a reference gene is not chosen adequately.

**Results:**

In the present study, we compared expression levels of five putative reference genes (HPRT1, ACTB, GAPDH, RPL13A and B2M) in primary cultures of four different human cells: mesenchymal stromal cells obtained from bone marrow, adipose tissue or umbilical cord Whartońs Jelly, and dermal fibroblasts, under different expansion and differentiation conditions. We observed that reference genes are not the same for different cells under the same culture conditions.

**Conclusion:**

Most stable reference genes under our experimental conditions were: RPL13A for adipose tissue- and Whartońs Jelly-derived mesenchymal stromal cells, and HPRT1 for bone marrow-derived mesenchymal stromal cells and dermal fibroblasts. ACTB was the most unstable gene when evaluating adipose tissue- and Whartońs Jelly-derived mesenchymal stromal cells, whilst GAPDH and B2M were the most unstable genes for bone marrow-derived mesenchymal stromal cells and dermal fibroblasts, respectively.

## Introduction

Real time polymerase chain reaction (PCR) was first described by Higuchi and coworkers in 1992 and the same group reported the first quantitative real time PCR the following year [1], [2]. Quantitative real time PCR (qPCR) is a widely used technique for quantification of messenger ribonucleic acids (mRNA) expression, not only in research but also in diagnostics. For a long time, a few genes, especially genes involved in basal metabolism such as beta-actin (ACTB) and glyceraldehyde 3-phosphate dehydrogenase (GAPDH) or ribosomal RNA, were believed to be constantly expressed in different cell lines, physiological situations and culture conditions. Such genes were routinely used as reference genes in qPCR, for improving data reliability since they exclude sample to sample variations and RNA quality and sample loading differences. By definition, an ideal reference gene should be expressed without variations in the different conditions studied. Only in 2000 Schmittgen and Zakrajsek published the first article about validation of reference genes [3]. Since then, importance of choosing the right reference gene in qPCR is growing quickly, as demonstrated by the increasing number of publications emphasizing that reference gene validation under new experimental conditions is an essential first step prior to any qPCR. It is clear today that there is no single reliable reference gene and that subtle modifications in experimental conditions, even when the same cells are being studied, have a deep effect on gene expression monitoring.

Human mesenchymal stromal cells (MSC) were first isolated by Friedenstein from a human bone marrow [4], [5]. Since then, MSC were also isolated from several other tissues such as adipose tissue [6], umbilical cord [7], dental pulp [8], synovial membrane [9] and placenta [10]. MSC from different sources are nowadays being tested in pre-clinical and clinical trials for a variety of diseases: in stroke, intravenously injected bone marrow-derived MSC (BM-MSC) were safe and improved recovery in a randomized trial including 52 patients [11]; allogeneic BM- MSC were also injected in patients with refractory acute graft-versus-host disease and the therapy resulted safe and effective, increasing overall survival rate from 44% (control group) to 80% (MSC-treated group) [12]; pre-conditioned autologous BM-MSC were safe and improved heart functionality and quality of life after endoventricularly injection in a multicenter randomized clinical trial involving patients with chronic heart failure [13]. Even when MSC were obtained from a variety of tissues, these examples clearly demonstrated that BM-MSC are the preferred cell type for clinical therapies. Cells obtained from different sources have different properties that are increasingly studied *in vitro* in the last years [14]–[16] but much information is still missing.

MSCs are defined by their cell surface markers (positive for CD105, CD73 and CD90 and negative for hematopoietic markers), their capacity to adhere to plastic surfaces and the ability to differentiate into adipocyte-, osteoblast- and chondrocyte-like cells under appropriate culture conditions [17]. This differentiation is mainly determined visually by staining with Oil Red [18], Alizarin Red [19] and Alcian Blue [20], respectively, but these methods are only qualitative and positive at late stages of differentiation. Quantification of different levels of differentiation would be useful in order to optimize cell differentiation conditions, to study differentiation kinetics or to compare cell culture supplements or cell types.

Another human cell type used in clinical therapies is dermal fibroblast. Reports from cultured human dermal fibroblasts can be found in the literature since the 1960s [21] but its first clinical application was approved by the Food and Drug Administration in USA only in 2011. Human autologous fibroblasts are used in aesthetic treatments for improving appearance of nasolabial fold wrinkles [22] and new applications are being tested [23]. *In vitro* studies suggest that fibroblasts share their immunoregulatory potential with MSC [24], [25], but they differ in their pro-angiogenic and anti-inflammatory abilities [26]. These facts make dermal fibroblasts an interesting alternative for regenerative medicine, since they are easy to obtain and their efficacy and safety have been already proven.

Human cell manipulation for clinical applications is nowadays performed using a variety of animal-derived supplements, such as trypsin, fetal calf or bovine serum and collagenases. Patients, researchers, companies and regulatory agencies are conscious of the risks that these materials introduce into cell culture and consequently into clinical therapies. All animal-derived supplements used during *ex vivo* cell expansion should be ideally avoided during the whole production process [27]–[29]. Fetal bovine serum (FBS) is maybe the main threat, since it is used at high concentrations during the whole procedure. A solution would be FBS substitution by human derivatives, such as plasma [30], [31] or platelet-rich plasma (PRP) [32].

Putative reference genes evaluated in this study were hypoxanthine phosphoribosyltransferase 1 (HPRT1), 60S ribosomal protein L13A (RPL13A), beta-2 microglobulin (B2M), GAPDH and ACTB (Table 1). These genes are commonly used as reference genes and they are appropriate for this kind of analysis since they are not functionally related, and therefore not co-regulated. GAPDH is a 37-kDa homotetrameric enzyme that catalyzes the oxidative phosphorylation of glyceraldehyde 3-phosphate in 1,3-bisphosphoglycerate, NAD+ and inorganic phosphate, a step of glycolysis but also involved in other non-metabolic processes, like apoptosis and axonal transport. HPRT1 is a 25-kDa enzyme that mediates guanine conversion into guanosine monophosphate and hypoxanthine into inosine monophosphate, playing a central function in purine nucleotides generation. RPL13A is a cytoplasmic 16-kDa protein that together with structural proteins and RNA molecules composed the 60 S ribosomal subunit. ACTB is a 41.7-kDa cytoskeletal protein that belongs to a family of highly conserved proteins mainly involved in cell structure and motility, muscle contraction and vesicles and organelles transport. B2M is a 13.7-kDa serum protein involved in antigen presentation; it is a component of the class I major histocompatibility complex and is found on the surface of all nucleated cells.

**Table 1:** Putative reference genes chosen in this study.

Gene	Name	Function	Amplicon (base pair)	RefSeq
ACTB	b-actin	Cell motility, structure, and integrity	139	NM_001101.3
GAPDH	glyceraldehyde 3-phosphate dehydrogenase	Carbohydrate metabolism	93	NM_002046.4 andNM_001256799.1
RPL13A	60S ribosomal protein L13A	Component of the 60S subunit of ribosome	81	NM_012423.2
B2M	beta-2 microglobulin	Associated to MHC I, antigen presentation	81	NM_004048.2
HPRT1	hypoxanthine phosphoribosyltransferase 1	Purine nucleotides synthesis throughthe purine salvage pathway	82	NM_000194.2

The main goal of the present study was to determine appropriate reference genes for 4 different primary cell cultures under expansion and differentiation conditions with 2 different culture supplements (PRP and FBS), aiming to characterize the effects of FBS substitution by an non-animal supplement. We studied expression of a set of 5 putative reference genes and we determined their expression stability using 4 different methods.

## Materials and Methods

### Ethics Statements

All the experimental procedures were approved by Ethics Research Committees. All donors signed an informed consent and all material was collected after the corresponding committee approval. Adipose tissue-derived MSC (AT-MSC) were obtained from abdominal liposuction during plastic surgery (approval number: 55219/12 - Ethics Research Committee of the Pro-Cardíaco Hospital, Rio de Janeiro, Brazil). Wharton’s Jelly-derived MSC (WJ-MSC) were obtained from full-term births (approval number: 336/10 - Ethics Research Committee of the Pro-Cardíaco Hospital, Rio de Janeiro, Brazil). Bone marrow-derived MSC (BM-MSC) were purified from remaining bone marrow obtained from posterior iliac crest of patients treated from nonunion fractures under clinical trial (approval number: 473/12 - Ethics Research Committee of the Pro-Cardíaco Hospital, Rio de Janeiro, Brazil). Human dermal fibroblasts (DF) were obtained from biopsies of 2 cm^2^ from donors’ normal skin, during a plastic surgery (approval number: 826/09, Ethics Research Committee of the Clementino Fraga Filho University Hospital, Rio de Janeiro, Brazil). Platelet rich plasma was prepared from human blood obtained from voluntary donors (approval number: 70649/12 - Ethics Research Committee of the Pro-Cardíaco Hospital, Rio de Janeiro, Brazil).

### Platelet-Rich Plasma (PRP) preparation

PRP was prepared as previously reported [33]. Briefly, blood was collected in tubes containing acid citrate and dextrose (ACD-tubes, BD, #364606) and centrifuged during 5 minutes at 300 *g*. The platelet-containing plasma above the buffy coat was separated and centrifuged during 17 minutes at 700 *g*. Pelleted platelets were suspended in a smaller volume of plasma and were activated by adding calcium chloride (20 mM). After incubating at 37°C during one hour and at 4°C during 16 hours, activated PRP was recovered by centrifugation at 3.000 *g* during 20 minutes at 18°C. Activated PRP, hereafter referred as PRP, was frozen at −20°C until use.

### Cell Isolation and Culture

#### BM-MSC

Nucleated cells were separated using Ficoll-Paque™ PLUS (GE Healthcare, #17-1440-02) by density gradient centrifugation at 700 *g* during 15 minutes. Isolated cells were washed with phosphate-buffered saline (PBS - LGC, #13-30259-05) and plated in T25 flasks with Minimum Essential Medium Eagle alpha (α-MEM; LGC, BR30007-05) supplemented with 10% FBS (LGC, #10-BIO-500) and ciprofloxacin (Sigma Aldrich, #17850) at 10 µg/mL. After 3 days in culture, monolayer was washed twice with PBS in order to remove non-adherent cells and culture medium was changed.

#### AT-MSC

Adipose tissue was washed 3 times with PBS in order to eliminate blood cells. Washed tissues were treated with 1.76 mg collagenase type I (Sigma, C9891) per tissue gram, during 30 minutes at 4°C and 30 minutes at 37°C with agitation. The associated proteolytic activity was inhibited by adding 1 volume Dubelccós Modified Eagle Medium (DMEM; LGC, #BR30002.05) supplemented with 10% FBS. After centrifugation (700 *g*, 7 minutes), pelleted cells were suspended in α-MEM supplemented with 10% FBS and plated in T25 flasks.

#### WJ-MSC

Umbilical cord was washed 3 times using PBS to exclude blood cells. After removal of blood vessels, Wharton’s jelly was cut into small pieces and digested with collagenase type II (Sigma, C6885), using 0.9 mg per tissue gram. After agitated incubation at 37°C during 1 hour, DMEM supplemented with 10% FBS was added. Cells were centrifuged at 700 *g* during 7 minutes and plated in T25 flasks with α-MEM supplemented with 10% FBS.

#### DF

Dermal tissue samples were isolated from freshly collected skin. Blood cells were removed by washing 3 times with PBS. Dermis and epidermis, cut in small pieces, were treated with 3 mg of collagenase type II per tissue gram. After agitated incubation during 1.5 hour at 37°C, DMEM supplemented with 10% FBS was added. Cells were washed and plated in T25 flasks with Roswell Park Memorial Institute medium (RPMI; LGC, BR30197.05) supplemented with 10% FBS.

Culture media were refreshed twice a week. When cells reached 75% confluence, they were detached using a 0.125% trypsin solution (Gibco, #27250-018), diluted and plated into new T flasks. Cells were sub-cultured up to the third passage and cryopreserved. An inverted microscope Eclipse TS-100 (Nikon) was used for monitoring cell cultures and photodocumentation was performed at 20× using an Opticam camera system.

For all experiments, pools of the different cell types were prepared by mixing equal number of cells from 4 donors in the same passage number. Cells used in this study were not cultured for more than 5 passages.

### Flow Cytometry

Cells were harvested using a 0.125% trypsin solution, washed with PBS and re-suspended in PBS containing 2% FBS. Viable and total cell numbers were determined using Trypan blue in Neubauer chambers. The following monoclonal antibodies were used as indicated by the manufacturer (BD Pharmingen®): CD90-PE (BD, #555596), CD73-FITC (BD, #561254), CD105-FITC (BD, #561443), CD45-FITC (BD, #347463), CD14-PE (BD, #555398) and CD34-PEcy5 (BD, #561819). At least 20,000 events were acquired on a BD FACSCalibur® flow cytometer and data was analyzed using CellQuest™ software.

### Differentiation in vitro

Cells were cultured in the corresponding culture media, supplemented with 10% PRP or 10% FBS, during 7 days, prior to differentiation induction. PRP concentration was reduced to 1% during differentiation in order to control cell growth. Low-glucose (LG) DMEM (LGC, BR30002.05) was used for all differentiation media, supplemented with a penicillin/streptomycin solution (LGC, BR30110-01) at 100 U/mL and 100 µg/mL, respectively. Working volume was 2 mL/well for 6-well plates (Corning, #3516) and 1 mL/well for 24-well plates (Corning, #3527). After 15–21 days, cells from 2 wells were detached, quantified by counting using Trypan blue in Neubauer chambers, washed and cryopreserved in RNAprotect cell reagent (QIAGEN, #76526) at −80°C until RNA extraction. Cells in another well were stained to visually confirm differentiation.

#### Adipogenic differentiation

cells were seeded into 6-well plates at 7,500 cells/cm^2^. Adipogenic medium consisted of LG-DMEM supplemented with 10% FBS or 1% PRP, 1 µM dexamethasone (Sigma, D4902), 0.5 mM 3-Isobutyl-1-methylxanthine (Sigma, I7018), 10 µM human insulin (Humulin-N) and 0.2 mM indomethacin (Sigma, I7378). Medium was changed twice a week. Cells were fixed in formalin buffer, washed with PBS and stained with 0.5% Oil Red O solution (Sigma, O0625) to confirm differentiation.

#### Osteogenic differentiation

10,900 cells/cm^2^ were seeded into 6-well plates. Osteogenic medium was composed of LG-DMEM supplemented with 10% FBS or 1% PRP, 10 nM dexamethasone (Sigma, D4902), 10 mM β-glycerophosphate (Calbiochem, #35675) and 50 µM L-ascorbic acid 2-phosphate (Sigma, A8960). Differentiation medium was changed twice a week. Cells were fixed and stained with 1% Alizarin Red S solution (Sigma, A5533), pH 4.2.

#### Chondrogenic differentiation

cells were seeded into 24-well plates as pellets containing 10^5^ cells. After 24 hours, chondrogenic medium was added: LG-DMEM supplemented with 1% FBS or 1% PRP, 50 µg/mL L-ascorbic acid 2-phosphate (Sigma, A8960), 10 ng/mL transforming growth factor-β3 (Sigma, SRP3171), 0.169 UI/mL human insulin and 6.25 µg/mL human transferrin (Sigma, T8158). After three weeks, the cells were fixed and stained with 1% toluidine blue solution (Sigma, #89640) to confirm chondrogenic differentiation.

Control cultures were maintained in parallel and control medium consisted of LG-DMEM supplemented with 10% FBS or 1% PRP and a penicillin/streptomycin solution at 100 U/mL and 100 µg/mL, respectively, for all three differentiation assays.

### RNA Extraction and qPCR

RNA was purified using the RNeasy Plus Mini kit (QIAGEN, #74134), according to the manufactureŕs instructions. RNA concentration was determined using a Nanodrop 2000 UV-Vis spectrophotometer (Thermo) and 350 ng RNA were reverse transcripted into complementary DNA (cDNA) using SuperScript VILO Mastermix (Invitrogen, #11755250) in a total reaction volume of 20 µL, following manufactureŕs protocol. A Verity Thermal Cycler (Applied Biosystems) was programmed as follows: 10 minutes at 25°C, 60 minutes at 42°C and 5 minutes at 85°C. cDNA was stored at −20°C until use. A total of 8 RNA samples were obtained for each cell pool: FBS- (1) and PRP-cultured undifferentiated cells (2), identified as day 0 (d0); cells induced to differentiate into the adipogenic phenotype in FBS (3) or PRP (4) supplemented media; cells cultured in osteogenic media containing FBS (5) or PRP (6) and chondrogenic-induced cells under FBS (7) or PRP (8) supplementation.

Oligonucleotides and probes for qPCR were purchased from Applied Biosystems (TaqMan gene expression assay, #4331182): GAPDH (Hs02758991_g1), HPRT1 (Hs02800695_m1), RPL13A (Hs03043885_g1), B2M (Hs00984230_m1) and ACTB (Hs03023880_g1). More information on these putative reference genes can be found in Table 1. qPCR reactions were performed in a Applied Biosystems 7500 Fast Real Time PCR System in a 20 µL reaction volume using TaqMan Gene Expression Mastermix (Applied Biosystems, #4369510), according to manufactureŕs instructions.

### Evaluation of Gene Stability and Statistical Analysis

Expression stability was evaluated using 3 softwares, based on different algorithms (geNorm, BestKeeper and NormFinder) and also using a statistical approach.

The Excel-based tool BestKeeper was developed by Pfaffl and colleagues [34] and is based in the fact that most stably expressed gene exhibits lowest quantification cycle (Cq) variation when cDNA input is constant. Cq values are used as input data and several parameters are calculated: geometric (GM) and arithmetic mean (AM) and different values expressing data variation: minimum and maximum Cq, Cq standard deviation (SD) and coefficient of variation (CV) and also ratio between minimum Cq and maximum Cq related to the geometric mean, together with its standard deviation, all three expressed as x-fold ratio. Authors determined that Cq values of a particular gene that varied in a way the produces a SD higher than 1 are considered inconsistent. They also suggest using more than just one reference gene in order to get more reliable results and parameters for this combination, called BK (BestKeeper), are calculated in the last table columns: BK is calculated for n = 5 when all 5 putative reference genes evaluated are stably expressed, and BK is calculated for a lower n when one o more putative reference genes are unstably expressed and therefore eliminated for parameter calculation.

GeNorm was developed in 2002 by Vandesompele and coworkers [35]. It determines pairwise variation among all putative reference genes and generates an M value, called gene-stability measurement, a dimensionless parameter that results from calculating the average of all logarithms of expression ratios between a putative gene and all other reference genes being evaluated. Lower M values correspond to more stably expressed genes.

Andersen and colleagues developed NormFinder, a model-based approach that generates a dimensionless stability value combining intra- and inter-group variation, using log-transformed Cq data [36]. NormFinder provides a ranking of all the evaluated genes, giving a stability value that is low when gene stability is higher. NormFinder needs at least three reference gene candidates and a minimum of 8 samples. Calculations and advantages over other methods, like the pair wise comparison, are described in details by the authors [36]. They emphasize that this model-based approach is not affected by co-regulation of putative reference genes and therefore, results are more robust.

Statistical approaches were already described in the literature [37]; it is based on the same fundaments as the BestKeeper software, but in our study was performed manually using Prism 5.00 Software (GraphPad Software Inc.).

## Results and Discussion

### Cell Isolation and Cultures

**Figure 1:**
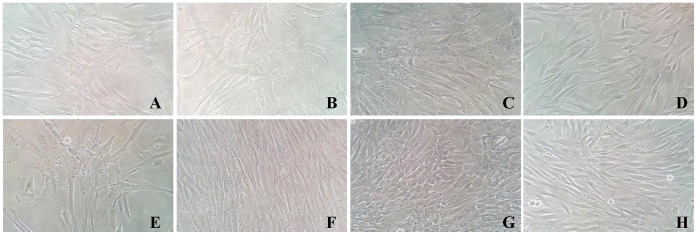
Cell culture morphology. BM-MSC in media containing 10% FBS (a) and 10% PRP (e); AT-MSC in media containing 10% FBS (b) and 10% PRP (f); WJ- MSC in media containing 10% FBS (c) and 10% PRP (g); DH in media containing 10% FBS (d) and 10% PRP (h). Cells were monitored using an Eclipse TS-100 inverted microscope (Nikon) and photodocumentation was performed at 20× using an Opticam camera system.

All cells obtained from different donors and pools generated thereof grew with viabilities above 95%, showing expected duplication times. Fibroblast-like morphology were observed in all cell pools (Figure 1). Cell identity was confirmed by adherence to plastic, cell surface markers expression and differentiation assays. All pools were positive (>95.0%) for mesenchymal cell markers (CD90, CD73 and CD105) and negative (<3%) for hematopoietic markers (CD45, CD14 and CD34). Differentiation was confirmed by staining as described in Materials and Methods (data not shown).

### RNA Purification

RNA concentration and purity are shown in Table S1. Absorbance values at 230, 260 and 280 nm were determined (A_230,_ A_260_ and A_280_, respectively). Mean A_260_/A_280_ value was 2.1±0.1, meaning there was no protein contamination; mean A_260_/A_230_ value was 1.8±0.4 confirming that reagents added during the purification procedure were completely washed out. For all samples, the amount of RNA obtained from 10^6^ cells was inside the range of RNA content in mammalian cells (10–30 µg RNA/10^6^ cells).

### Reference Gene Selection

Reference gene selection relies on pippeting reproducibly same cDNA amounts of different samples and subsequent quantification of gene expression: expression stability is reflected in Cq value stability through the samples. Reproduction of Cq values for sample replicates inside a plate (biological replicates or intra-assay variation) or between different plates performed sometimes even by different researchers (technical replicates or inter-assay variation) can be compared using coefficient of variation (CV). Acceptable CV values for biological replicates can go up to 30% but CV values up to 50% are also accepted for qPCR results [38].

CV was calculated for Cq values obtained for all 4 different cell pools and all 5 putative reference genes as intra- and inter-assay values. Intra-assay CV varied from 0.16 to 1.68 for AT-MSC, from 0.04 to 1.78 for WJ-MSC, from 0.05 to 3.59 for BM-MSC and from 0.18 to 6.38 for DF. These values are low, indicating that Cq is highly reproducible for biological replicates. Inter-assay CV range was 6.50–19.04 for AT-MSC, 6.96–11.62 for WJ-MSC, 2.91–15.29 for BM-MSC and 2.73–6.12 for DF. CV values are usually higher for inter-assay comparisons, because of operator and day variations, but our values are still low, again confirming high reproducibility of our experiments.

### Bestkeeper

Results obtained using the BestKeeper software for determining suitable reference genes are shown in Table 2 (AT-MSC), Table 3 (WJ-MSC), Table 4 (BM-MSC) and Table 5 (DF). For WJ-MSC and DF, all genes showed an SD value lower than 1, meaning their expression was stable among all 8 conditions studied and consequently all of them can be used as reference genes. The lowest SD values were obtained for RPL13A (0.35) and B2M (0.49) for WJ-MSC and B2M (0.59) and RPL13A (0.66) for DF.

**Table 2:** BestKeeper results obtained for adipose tissue-derived mesenchymal stromal cells.

AT-MSC (n = 8)	B2M	RPL13A	HPRT1	GAPDH	ACTB	BK (n = 5)	BK (n = 4)
GM [Cq]	24.58	22.86	31.57	23.47	24.11	25.14	25.40
AM [Cq]	24.59	22.87	31.57	23.47	24.31	15.15	25.40
Min [Cq]	23.89	22.37	30.54	23.07	19.63	24.30	25.05
Max [Cq]	25.15	23.28	32.29	23.73	31.14	26.51	25.67
SD [± Cq]	0.35	0.25	0.44	0.20	2.33	0.49	0.15
CV [% Cq]	1.43	1.10	1.38	0.83	9.60	1.94	0.58
Min [x-fold]	−1.61	−1.41	−2.04	−1.32	−22.40		
Max [x-fold]	1.48	1.34	1.66	1.20	130.70		
SD[± x-fold]	1.28	1.19	1.35	1.15	5.04		

The last column (BK, n = 4) shows the parameters obtained after combining all stably expressed genes (B2M, RPL13A, HPRT1 and GAPDH), so they can be use as a single normalization factor. n: number of samples; BK: BestKeeper; GM [Cq]: geometric mean of Cq; AM [Cq]: arithmetic mean of Cq; Min [Cq]: minimum value of Cq; Max [Cq]: maximum value of Cq; SD [± Cq]: standard deviation of the Cq; CV [% Cq]: coefficient of variation expressed as a percentage on the Cq level; Min [x-fold]: minimum value of expression levels expressed as an absolute x-fold; Max [x-fold]: maximum value of expression levels expressed as an absolute x-fold; SD [± x-fold]: standard deviation of the absolute regulation coefficients. SD value obtained for ACTB (2.33) is higher than cutoff (1.0), so ACTB was defined as an unstably expressed gene and therefore was not considered for BK (n = 4) calculations.

**Table 3:** BestKeeper results obtained for Whartońs Jelly-derived mesenchymal stromal cells.

WJ-MSC(n = 8)	B2M	RPL13A	HPRT1	GAPDH	ACTB	BK (n = 5)
GM [Cq]	23.94	22.76	26.92	23.65	24.22	24.26
AM [Cq]	23.95	22.76	26.94	23.67	24.23	24.27
Min [Cq]	23.02	21.78	25.69	22.43	22.48	23.27
Max [Cq]	24.91	23.28	28.66	25.13	25.22	25.15
SD [± Cq]	0.49	0.35	0.84	0.81	0.78	0.61
CV [% Cq]	2.04	1.52	3.11	3.42	3.23	2.53
Min [x-fold]	−1.90	−1.97	−2.35	−2.33	−3.33	
Max [x-fold]	1.95	1.44	3.32	2.78	2.01	
SD [± x-fold]	1.40	1.27	1.79	1.75	1.72	

n: number of samples; BK: BestKeeper; GM [Cq]: geometric mean of Cq; AM [Cq]: arithmetic mean of Cq; Min [Cq]: minimum value of Cq; Max [Cq]: maximum value of Cq; SD [± Cq]: standard deviation of the Cq; CV [% Cq]: coefficient of variation expressed as a percentage on the Cq level; Min [x-fold]: minimum value of expression levels expressed as an absolute x-fold; Max [x-fold]: maximum value of expression levels expressed as an absolute x-fold; SD [± x-fold]: standard deviation of the absolute regulation coefficients. No unstably expressed gene detected.

**Table 4:** BestKeeper results obtained for bone marrow-derived mesenchymal stromal cells.

BM-MSC (n = 8)	B2M	RPL13A	HPRT1	GAPDH	ACTB	BK (n = 5)	BK (n = 3)
GM [Cq]	24.98	22.51	28.95	23.57	24.79	24.87	25.34
AM [Cq]	24.99	22.52	28.96	23.60	24.86	24.88	25.35
Min [Cq]	24.10	22.04	27.07	21.35	23.06	23.88	24.35
Max [Cq]	25.80	23.52	30.17	24.91	29.32	26.30	26.00
SD [± Cq]	0.57	0.39	0.62	1.03	1.42	0.61	0.44
CV [% Cq]	2.27	1.75	2.15	4.34	5.70	2.47	1.72
Min [x-fold]	−1.84	−1.39	−3.67	−4.68	−3.32		
Max [x-fold]	1.77	2.01	2.33	2.52	23.00		
SD [± x-fold]	1.48	1.31	1.54	2.04	2.67		

The last column (BK, n = 3) shows the parameters obtained after combining all stably expressed genes (B2M, RPL13A and HPRT1), so they can be use as a single normalization factor. n: number of samples; BK: BestKeeper; GM [Cq]: geometric mean of Cq; AM [Cq]: arithmetic mean of Cq; Min [Cq]: minimum value of Cq; Max [Cq]: maximum value of Cq; SD [± Cq]: standard deviation of the Cq; CV [% Cq]: coefficient of variation expressed as a percentage on the Cq level; Min [x-fold]: minimum value of expression levels expressed as an absolute x-fold; Max [x-fold]: maximum value of expression levels expressed as an absolute x-fold; SD [± x-fold]: standard deviation of the absolute regulation coefficients. SD values obtained for ACTB (1.42) and GAPDH (1.03) are higher than cutoff (1.0), so both genes were defined as unstably expressed genes and therefore were not considered for BK (n = 3) calculations.

**Table 5:** BestKeeper results obtained for dermal fibroblasts.

DF (n = 8)	B2M	RPL13A	HPRT1	GAPDH	ACTB	BK (n = 5)
GM [Cq]	22.86	21.63	30.41	21.62	20.13	23.07
AM [Cq]	22.87	21.64	30.42	21.64	20.15	23.08
Min [Cq]	21.96	20.65	28.77	20.46	18.50	22.26
Max [Cq]	24.11	22.78	31.51	22.87	21.52	24.33
SD [± Cq]	0.59	0.66	0.71	0.78	0.80	0.48
CV [% Cq]	2.59	3.03	2.32	3.62	3.95	2.07
Min [x-fold]	−1.86	−1.97	−3.10	−2.24	−3.08	
Max [x-fold]	2.39	2.23	2.15	2.37	2.63	
SD [± x-fold]	1.51	1.57	1.63	1.72	1.74	

The last column (BK, n = 5) shows the parameters obtained after combining all stably expressed genes (B2M, RPL13A and HPRT1), so they can be use as a single normalization factor. n: number of samples; BK: BestKeeper; GM [Cq]: geometric mean of Cq; AM [Cq]: arithmetic mean of Cq; Min [Cq]: minimum value of Cq; Max [Cq]: maximum value of Cq; SD [± Cq]: standard deviation of the Cq; CV [% Cq]: coefficient of variation expressed as a percentage on the Cq level; Min [x-fold]: minimum value of expression levels expressed as an absolute x-fold; Max [x-fold]: maximum value of expression levels expressed as an absolute x-fold; SD [± x-fold]: standard deviation of the absolute regulation coefficients. No unstably expressed gene detected.

Regarding AT-MSC and BM-MSC, they had at least one gene than was unstably expressed under our experimental conditions: ACTB (SD = 2.33) for AT-MSC samples and GAPDH (SD = 1.03) and ACTB (SD = 1.42) for BM-MSC. The most stable genes expressed in AT-MSC were GAPDH (SD = 0.20) and RPL13A (SD = 0.25); for BM-MSC, were RPL13A (SD = 0.39) and B2M (SD = 0.57). For these two MSC, the software calculates a new BestKeeper index, resulting from the combination of those stably expressed genes. Pfaffl and coworkers suggest that a combination of appropriate reference genes would generate more reliable results than using a single gene [34].

### geNorm

Table 6 shows the stability M values generated by the geNorm software. Hellemans and coworkers stated that stably expressed reference genes show M values lower than 0.5, but for heterogeneous sample panels M values up to 1 are accepted [39]; they included in the heterogeneous sample definition treated cell culture, that would be our case.

**Table 6:** geNorm M stability values for all 4 cell types, considering stable expression throughout all 8 samples.

	DF	AT-MSC	BM-MSC	WJ-MSC
B2M	0.935	0.634	0.632	0.666
RPL13A	0.851	0.483	0.553	0.620
HPRT1	0.605	0.430	0.470	0.601
GAPDH	0.648	0.451	0.516	0.595
ACTB	0.699	1.120	0.722	0.691

Only for AT- and BM-MSC samples some M values were lower than 0.5; the most stably expressed genes were HPRT1, GAPDH and RPL13A. Considering acceptable values as high as 1, only ACTB for AT-MSC should be excluded and considered to be a highly unstable reference gene.

The most stable genes for WJ-MSC were GAPDH, HPRT1 and RPL13A; for BM-MSC, HPRT1, GAPDH and RPL13A; for AT-MSC, HPRT1, GAPDH and RPL13A; and for DH, HPRT1, GAPDH and ACTB. The most unstable gene was ACTB for all 3 MSC and B2M for DF.

### Normfinder

Results obtained using NormFinder software are shown in Table 7. Best reference genes were GAPDH for WJ-MSC and HPRT1 for DF; for both cell types B2M was the most unstable gene. For AT- and BM-MSC, standard error was higher than the stability value itself, especially for stably expressed reference genes, making results less reliable; this was also reported by the software authors in their own results [36]. The most unstable genes were ACTB for AT-MSC and ACTB and B2M for BM-MSC. Due to high standard errors, it was not possible to determine one single stably expressed gene for both cell types using NormFinder.

**Table 7:** NormFinder stability results; higher stability values mean unstable expression under evaluated conditions.

	Gene name	Stability value	Standard error
AT-MSC	B2M	0.267	0.458
	RPL13A	0.288	0.443
	HPRT1	0.659	0.377
	GAPDH	0.297	0.437
	ACTB	3.396	0.911
WJ-MSC	B2M	0.684	0.202
	RPL13A	0.422	0.156
	HPRT1	0.581	0.181
	GAPDH	0.201	0.162
	ACTB	0.461	0.161
BM-MSC	B2M	3.592	0.985
	RPL13A	0.337	0.895
	HPRT1	0.337	0.895
	GAPDH	0.338	0.894
	ACTB	2.296	0.704
DF	B2M	0.865	0.261
	RPL13A	0.796	0.248
	HPRT1	0.457	0.202
	GAPDH	0.593	0.214
	ACTB	0.575	0.212

### Statistical Analysis

Statistical approaches to identify reference genes were already described in the literature [37]. Q-values were obtained after pair-wise comparison of each Cq value with the one obtained for the same cell pool cultured in FBS and not induced (sample 1). AM and SD for each gene and cell line combination were compared; genes showing the smallest mean Q-value were the most stable, as same initial RNA amounts were used in all the experiments and therefore low Cq variations are expected. Results are shown in Table 8. HPRT1 was the most stably expressed reference gene for DF, and RPL13A for WJ-MSC. Results obtained for AT- and BM-MSC showed high standard deviation values, as obtained with NormFinder software. Judging by the arithmetic average, ACTB and GAPDH were the most unstable reference genes for AT- and BM-MSC, respectively, but due to high standard errors, these results cannot be confirmed.

**Table 8:** Q-values (arithmetic mean and standard deviation) obtained after statistical pair wise comparisons.

	AT-MSC		WJ-MSC		BM-MSC		DF	
	AM	SD	AM	SD	AM	SD	AM	SD
B2M	2.1	1.1	4.9	4.0	6.2	6.1	8.7	7.0
RPL13A	1.4	0.7	2.5	1.4	2.9	2.6	6.6	3.7
HPRT1	3.0	1.6	4.7	3.1	2.4	2.1	1.2	0.9
GAPDH	2.6	1.0	7.9	5.1	10.8	4.4	4.2	2.4
ACTB	22.1	21	12.2	4.4	6.2	6.4	4.7	3.8

AM: arithmetic mean; SD: standard deviation.

### Data Interpretation

All the 4 methods applied for reference gene stability evaluation are based on stability values, where lower values mean higher stability. Summing up all values for each gene, we obtained a single value and therefore a final ranking considering all 4 different methods. Ranked genes for each cell line are shown in Table 9.

**Table 9:** Summary of results obtained for the different 4 evaluation methods.

	AT-MSC	WJ-MSC	BM-MSC	DF
most stable	RPL13A	RPL13A	HPRT1	HPRT1
	B2M	HPRT1	RPL13A	GAPDH
	GAPDH	B2M	ACTB	ACTB
	HPRT1	GAPDH	B2M	RPL13A
less stable	ACTB	ACTB	GAPDH	B2M

RPL13A was the most stably expressed gene for both AT-MSC and WJ-MSC, while HPRT1 was the most stable for BM-MSC and DF. The most unstably expressed genes were ACTB for AT-MSC and WJ-MSC, GAPDH for BM-MSC and B2M for DF.

There are several reports in the literature regarding reference gene selection for the same cell types here studied. Ragni and colleagues selected reference gene for FBS- supplemented AT-MSC under proliferation and tri-lineage differentiation circumstances; they concluded that RPL13A was the most stably expressed gene, followed by GAPDH and that B2M and ACTB were the most unstable mRNA [16]. Catalán and coworkers have shown that GAPDH is the most unstable gene for obesity studies, among 11 putative reference genes studied [40]. Fink and colleagues also concluded that GAPDH was unstably expressed under cell propagation, differentiation and hypoxic exposure [41]. GAPDH was for a long time considered the gold reference gene, even when this was not experimentally demonstrated [42]. Comparative studies on adipocytes have concluded that GAPDH was the most stable gene among 11 commonly used reference genes, which clearly shows that reference genes are closely related to experimental conditions [43]. All these reports confirmed that there is no single reliable reference gene for a cell type and that they depend on culture conditions.

For BM-MSC, Quiroz and coworkers studied GAPDH, RPL13A and ACTB as putative reference genes under osteogenic conditions and concluded that RPL13A and GAPDH were not regulated by osteogenic media, but ACTB was [44]. Another group also validated RPL13A as a reference gene for qPCR normalization under expansion, differentiation, different oxygen concentration and *in vivo* experiments [37], [45]. Ragni and colleagues concluded that B2M was the most unstable gene expressed in BM-MSC under culture conditions similar to ours [16].

Only a few reports on stably expressed genes for WJ-MSC are available. As described by Ragni and colleagues, GAPDH and B2M would be considered unstably expressed genes for WJ-MSC [16]. Wang and coworkers concluded that RPL13A was an appropriate reference gene for WJ-MSC at low passages and that this situation showed significant changes when increasing passage number [46].

Clear examples of using the wrong reference gene are shown by different researchers, who calculated expression levels of representative genes under differentiation conditions using suitable and unsuitable reference genes for data normalization, obtaining inconsistent results [16], [47]. Ragni and colleagues compared gene expression of differentiation markers when normalized with the most stable and the most unstable reference genes and clearly showed result distortion: normalization using stably expressed genes resulted in no gene regulation through all culture conditions studied, but normalization using unstably expressed genes resulted in a fake upregulated expression in a time-dependent manner [16]. Zhai and coworkers calculated gene expression of chondrogenic markers against 10 different reference genes, including stably expressed and commonly used reference genes (such as GAPDH and ACTB) and showed huge differences in expression results [47].

## Conclusions

In the present study, we established appropriate reference genes for 4 different cell lines (AT-, WJ-, BM-MSC and DF) under differentiation conditions in culture medium supplemented with FBS or PRP. We applied 4 different methods for determining appropriate reference genes. We observed that different softwares, based on different theories, did not give the same results, but combinations would result in more reliable data.

In conclusion, we have determined stably expressed mRNA molecules, which can be used as reference genes in qPCR studies when AT-, WJ-, BM-MSC and DF are cultured with FBS and PRP under adipogenic, osteogenic, chondrogenic and control conditions.

## Supporting Information

Table S1 **RNA concentration and quality for all samples.** DF: dermal fibroblast; AT-MSC: adipose tissue-derived mesenchymal stromal cell; WJ-MSC: Whartońs Jelly-derived mesenchymal stromal cell; BM-MSC: bone marrow-derived mesenchymal stromal cell; FBS: fetal bovine serum; PRP: platelet-rich plasma; d0: day 0; d15: day 15; d16: day 16; d17: day 17; d18: day 18; d19: day 19; d20: day 20; d21: day 21; A230: absorbance at 230 nm; A260: absorbance at 260 nm; A280: absorbance at 280 nm; A: cells cultured under adipogenic differentiation media; O: cells cultured under osteogenic differentiation media; C: cells cultured under chondrogenic differentiation media; RNA: ribonucleic acid.
(DOCX)
