# Supplementary material for: Identification of Appropriate Reference Genes for Human Mesenchymal Cells during Expansion and Differentiation
Source: PLoS One. 2013 Sep 2;8(9):e73792. doi: 10.1371/journal.pone.0073792 (PMC3759474; doi:10.1371/journal.pone.0073792)
Supplement: Table S1 — RNA concentration and quality for all samples. DF: dermal fibroblast; AT-MSC: adipose tissue-derived mesenchymal stromal cell; WJ-MSC: Whartońs Jelly-derived mesenchymal stromal cell; BM-MSC: bone marrow-derived mesenchymal stromal cell; FBS: fetal bovine serum; PRP: platelet-rich plasma; d0: day 0; d15: day 15; d16: day 16; d17: day 17; d18: day 18; d19: day 19; d20: day 20; d21: day 21; A230: absorbance at 230 nm; A260: absorbance at 260 nm; A280: absorbance at 280 nm; A: cells cultured under adipogenic differentiation media; O: cells cultured under osteogenic differentiation media; C: cells cultured under chondrogenic differentiation media; RNA: ribonucleic acid. (DOCX) [file pone.0073792.s001.docx]

| Sample (harverst time) | Cell number | RNA concentration (μg/mL) | A_260_/A_280_ | A_260_/A_230_ | μg RNA/10^6^ céls |
| --- | --- | --- | --- | --- | --- |
| DF SFB (d0) | 230,000 | 136.0 | 2.04 | 1.45 | 29.6 |
| DF PRP (d0) | 680,000 | 182.6 | 2.08 | 1.40 | 13.4 |
| DF SFB A (d16) | 510,000 | 162.7 | 2.11 | 1.81 | 16.0 |
| DF PRP A (d16) | 430,000 | 173.9 | 2.10 | 1.81 | 20.2 |
| DF SFB O (d16) | 950,000 | 338.8 | 2.05 | 2.09 | 17.8 |
| DF PRP O (d16) | 680,000 | 184.9 | 2.07 | 1.88 | 13.6 |
| DF SFB C (d17) | 190,000 | 52.4 | 2.12 | 1.53 | 13.8 |
| DF PRP C (d17) | 86,000 | 20.0 | 2.19 | 1.28 | 11.6 |
| AT-MSC SFB (d0) | 500,000 | 124.7 | 2.06 | 1.66 | 12.5 |
| AT-MSC PRP (d0) | 500,000 | 132.2 | 2.05 | 1.55 | 13.2 |
| AT-MSC SFB A (d18) | 275,000 | 72.3 | 2.02 | 0.81 | 13.1 |
| AT-MSC PRP A (d18) | 429,000 | 87.5 | 2.07 | 1.32 | 10.2 |
| AT-MSC SFB O (d18) | 763,750 | 342.3 | 2.03 | 1.76 | 22.4 |
| AT-MSC PRP O (d17) | 311,250 | 153.2 | 2.08 | 1.81 | 24.6 |
| AT-MSC SFB C (d17) | 31,000 | 6.3 | 2.36 | 2.14 | 10.2 |
| AT-MSC PRP C (d17) | 61,500 | 21.8 | 2.08 | 2.42 | 17.7 |
| BM-MSC SFB (d0) | 597,500 | 156.6 | 2.09 | 2.02 | 13.1 |
| BM-MSC PRP (d0) | 417,000 | 118.8 | 2.15 | 1.59 | 14.2 |
| BM-MSC SFB A (d21) | 107,250 | 32.4 | 2.09 | 1.70 | 15.1 |
| BM-MSC PRP A (d21) | 432,250 | 176.2 | 2.08 | 1.55 | 20.4 |
| BM-MSC SFB O (d20) | 140,000 | 39.9 | 2.10 | 1.66 | 14.3 |
| BM-MSC PRP O (d20) | 230,000 | 78.9 | 2.06 | 1.91 | 17.2 |
| BM-MSC SFB C (d19) | 15,000 | 7.0 | 2.05 | 2.21 | 23.3 |
| BM-MSC PRP C (d19) | 18,000 | 10.6 | 2.13 | 1.74 | 29.4 |
| WJ-MSC SFB (d0) | 1,470,000 | 326.1 | 2.04 | 1.54 | 11.1 |
| WJ-MSC PRP (d0) | 450,000 | 188.0 | 2.08 | 1.55 | 20.9 |
| WJ-MSC SFB A (d21) | 136,500 | 32.5 | 2.13 | 2.82 | 11.9 |
| WJ-MSC PRP A (d21) | 185,250 | 37.7 | 2.12 | 2.19 | 10.2 |
| WJ-MSC SFB O (d20) | 155,000 | 54.4 | 2.08 | 1.70 | 17.5 |
| WJ-MSC PRP O (d20) | 205,000 | 52.0 | 2.09 | 2.50 | 12.7 |
| WJ-MSC C SFB (d18) | 265,000 | 116.1 | 2.13 | 2.46 | 21.9 |
| WJ-MSC C PRP (d15) | 25,000 | 6.7 | 2.09 | 2.15 | 13.4 |
